# Submucosal Mitomycin C Injection in the Endoscopic Treatment of Laryngotracheal Stenosis: Experience of a Tertiary Center

**DOI:** 10.3390/jcm14228022

**Published:** 2025-11-12

**Authors:** Elena Russo, Luca Canali, Luca Cerri, Giuseppe Mangiameli, Umberto Cariboni, Giuseppe Marulli, Giuseppe Spriano, Giorgio Maria Ferraroli, Armando De Virgilio

**Affiliations:** 1 Department of Biomedical Sciences, Humanitas University, Via Rita Levi Montalcini, 4, 20090 Pieve Emanuele, MI, Italy; elena.russo198@gmail.com (E.R.);; 2 Otorhinolaryngology, Head & Neck Surgery Unit, IRCCS Humanitas Research Hospital, Via Manzoni 56, 20089 Rozzano, MI, Italy; 3 Department of ‘Organi di Senso’, University “Sapienza”, Viale Dell’ Università, 33, 00185 Rome, RM, Italy; 4 Thoracic Surgery Unit, IRCCS Humanitas Research Hospital, Via Manzoni 56, 20089 Rozzano, MI, Italy

**Keywords:** mitomycin C, laryngotracheal stenosis, subglottic stenosis, endoscopic surgery

## Abstract

**Background/Objectives**: To assess the safety and efficacy of submucosal mitomycin C (MMC) injection as an adjunct in patients undergoing endoscopic treatment for laryngotracheal stenosis (LTS). **Methods**: All patients affected by LTS receiving endoscopic treatment with adjuvant MMC were screened and selected from a retrospective database spanning from May 2022 to July 2023 at IRCCS Humanitas Research Hospital (Rozzano, Italy). Demographic data, bronchoscopic findings, and treatment-related outcomes were recorded and analyzed. **Results**: A total of 16 patients were included in the study. Subglottic stenosis was the most common condition, occurring in 88.9% of cases. All procedures were completed safely without intraoperative or postoperative complications or conversion to open surgery. Re-stenosis occurred in 6 (37.5%) cases, with a median time to recurrence of 136.5 (±43.9) days, requiring additional treatments. At last follow-up, all included patients were asymptomatic, except for two who reported mild dyspnea. **Conclusions**: Submucosal MMC injections represent a safe and promising delivery method in the endoscopic treatment of LTS and may provide a more durable effect on the stenosis reducing the risk of recurrence. Further prospective studies are needed to evaluate MMC’s effectiveness and develop standardized treatment protocols.

## 1. Introduction

Laryngotracheal stenosis (LTS) is a complex and challenging condition resulting in airway narrowing involving the trachea, larynx or both. Small variations in the diameter and length of the affected segment can produce substantial airflow limitation, sometimes progressing to critical respiratory compromise that necessitates urgent medical or surgical intervention [1]. In addition to airway obstruction, patients frequently experience dysphonia and dysphagia, reflecting the multifaceted impact of the disease on upper airway function [2].

The etiology of LTS is varied and multifactorial. Presently, the most common causes are iatrogenic, resulting from procedures such as intubation and tracheostomy [3]. Mechanical ventilation can cause mucosal injuries through ischemic damage from cuff pressure on the trachea or post-tracheostomy injury, or a combination of both [4]. The reported incidence of LTS following laryngotracheal intubation ranges from 6% to 21%, while post-tracheostomy LTS ranges from 0.6% and 21% [5]. Other causes include external trauma, autoimmune diseases, and tumors.

The management of LTS remains a challenging task, often requiring a spectrum of surgical approaches tailored to disease severity and patient characteristics [6]. Despite rigorous patient selection and multiple endoscopic or open reconstructive procedures, the recurrence of stenosis is common, reflecting the persistent aberrant wound-healing mechanisms responsible for the initial stenosis [7,8,9]. Recent reports confirm that restenosis may occur in up to 50% of cases following endoscopic dilation alone, underscoring the need for effective adjuvant therapies capable of modulating scar remodeling and prolonging airway patency [10]. The high rate of stenosis relapse has prompted increasing interest in pharmacologic modulation of the wound-healing response. Various adjuvant options have been investigated, including steroid injections, topical application of mitomycin C (MMC), topical heparin, 5-fluorouracil, and halofuginone [11,12,13]. MMC is a natural antibiotic derived from *Streptomyces caespitosus* [14]. In addition to its antibiotic properties, MMC acts as an antineoplastic and alkylating agent by interfering with DNA synthesis [15]. Its initial application can be traced back to 1963 when ophthalmologists achieved excellent results using it in pterygium surgery [16]. In 1998, Ingrams et al. [17] conducted research on the potential effects of MMC on the airways. In particular, they investigated its impact on the healing of paranasal sinus mucosa in rabbits, demonstrating improved antrostomy patency without significant disruption of the ciliated respiratory epithelium. While both animal and human studies have examined the utilization of MMC in the upper and lower airways [18,19,20,21,22,23], uncertainty persists due to the limited availability and heterogeneity of published data.

Although topical MMC application using pledgets is well documented and incorporated into many endoscopic protocols, clinical outcomes have remained inconsistent, largely due to variability in concentration, exposure time, and tissue penetration [20,21,22,23,24,25]. In this context, submucosal intralesional MMC injection is an underexplored delivery method which may achieve deeper, more uniform tissue distribution, potentially enhancing its antifibrotic activity while minimizing toxicity.

Through a retrospective collection of data on patients treated at our institution, we aim to evaluate the efficacy and safety of adjuvant submucosal MMC in the endoscopic treatment of laryngotracheal stenoses.

## 2. Materials and Methods

### 2.1. Study Population

A retrospective review of all patients affected by LTS receiving endoscopic treatment with adjuvant MMC from May 2022 until July 2023 in the division of thoracic surgery at IRCCS Humanitas Research Hospital (Rozzano, Italy) was performed. Inclusion criteria comprised: (1) diagnosis of non-malignant LTS confirmed by endoscopic examination; (2) treatment with bronchoscopic dilation, laser resection followed by adjuvant submucosal MMC injection at the time of enrolment (patients who had undergone previous or subsequent endoscopic treatments without MMC were not excluded); (3) availability of at least 6 months of follow-up data. Exclusion criteria included: (1) incomplete clinical records; (2) malignancy-related airway stenosis. All patients with documented LTS were evaluated with flexible fiberoptic bronchoscopy and computed tomography (CT) of the neck and chest, whenever deemed necessary to better characterize the site and severity of the stenosis. This study was performed following the principles of the Declaration of Helsinki and was approved by the Ethics Committee of Humanitas University on 19 September 2023 (ID number 3259). Informed consent was obtained for each patient.

### 2.2. Outcome Measures

The primary outcome of this study was treatment success, defined as the restoration of an endoscopically patent airway and improvement of respiratory symptoms following the index procedure, without intraoperative or postoperative complications;

Secondary outcomes included: (1) the occurrence of restenosis, defined as the recurrence of airway narrowing requiring additional intervention during follow-up (2) Time to recurrence, calculated from the date of the index procedure to the date of restenosis detection; and (3) Treatment safety by documenting procedure-related complications or adverse events.

### 2.3. Demographic Data

Patients’ baseline information, including their demographic details, clinical characteristics, and any pre-existing medical conditions, was collected upon their admission to the thoracic surgery division. Specific data related to the diagnosis of LTS (idiopathic or post-intubation/tracheostomy) was also documented. Additionally, information concerning COVID-19 infection status, the duration of intubation with invasive mechanical ventilation (in days), the method used for tracheostomy (percutaneous or surgical), the time taken for decannulation, and any instances of redo-tracheostomy, along with their respective durations, was recorded.

### 2.4. Flexible Bronchoscopy

Flexible bronchoscopy was systematically performed under topical anesthesia with 2% lidocaine. A fiberoptic laryngoscope (Fujifilm, Tokyo, Japan) was inserted nasally to assess the vocal cords and the posterior pharyngeal and subglottic areas. Any identified abnormalities were documented. The type, location, and severity of LTS detected through flexible bronchoscopy were described. The stenosis was categorized as either eccentric or concentric. All procedures were performed by an expert thoracic surgeon (G.F.).

### 2.5. Radiological Data

CT scans were conducted using a General Electric CT Scanner (HiSpeed CT/i, GE, Milwaukee, WI, USA) equipped with standard reconstruction software. The scanning protocol involved 3 mm transaxial collimation, commencing from the level of the hyoid bone and extending to the carina. A 20 cm field of view was utilized with standard interpolation and algorithm. The pitch was set between 1.0 and 1.5, with a 1.5 mm overlap. Furthermore, coronal and sagittal reconstructions were generated at 1.5 mm intervals using shaded surface reconstruction. To assess the degree of stenosis, we measured the most significant percentage of narrowing in the transaxial plane and validated these findings with coronal and sagittal reconstructions. Stenosis was defined as a reduction in the transaxial diameter exceeding 10%. If coronal or sagittal reconstructions indicated a more substantial stenosis than observed in transaxial views, we recorded the higher percentage stenosis. LTS severity was categorized into Grade 1 (luminal narrowing < 50%), Grade 2 (luminal narrowing ≥ 50% but <70%), and Grade 3 (luminal narrowing ≥ 70%) in accordance with the Myer-Cotton grading system [26], and further classified based on subsite (supraglottis, glottis, subglottis, trachea) involved, as described by the European Laryngological Society classification [27].

To determine stenosis length and the distance from the vocal cords, we applied the measurement methods proposed by Morshed et al. [28]. This involved using a program ruler to measure the maximal projectional length of laryngotracheal luminal narrowing. Radiological data collection was carried out by a single radiologist (C.G.), who was not involved in the patients’ surgical management and was unaware of their clinical symptoms.

### 2.6. Surgical Treatment

Patients were positioned supine and pre-oxygenated by high flow nasal-cannula oxygen therapy (HFNO) at 100% fractional inspired oxygen (FiO_2_) administration; after general anesthesia by target-controlled infusion of propofol/remifentanil, patients were intubated through a rigid bronchoscope and ventilation was assured manually during the non-interventional time. Endoscopic magnification was achieved using a 6.2 mm diameter flexible bronchoscope (Fujifilm, Tokyo, Japan) with a 2.8 mm working channel, connected to a video monitor. The endoscopic procedures performed depended on the type of LTS and consisted of laser radial incision through the stenosis and/or tracheal dilatation by either rigid bronchoscopes of increasing sizes or tracheobronchial balloons. Laser incisions were performed under intermittent apnea conditions using the LOKKI Lis system (LOKKI, LOBEL Medical, Les Roches-de-Condrieu, France), which utilizes an Nd: YAP (Yttrium-Aluminum-Perovskite) laser emitting at a patented wavelength of 1340 nm. The laser beam was delivered through a 600-micron diameter optical fiber (CeramOptec GmbH, Bonn, Germany), operating in continuous pulse mode with an output power of 10 W. Typically, 3 to 4 radial incisions were performed. Balloon dilations were conducted with the Micro-Tech Disposable Multistage Dilation Balloon Catheter (Micro-Tech Co., Ltd., Nanjing, China). This catheter had a length of 180 cm and a width of 2.36 mm (7 F), along with a 55 cm-long balloon, a channel diameter exceeding 2.8 mm, and a 0.035-inch guidewire. Notably, this dilation balloon catheter is expressly engineered to generate three distinct diameters (12, 13.5, and 15 mm) at three different pressures (3, 4.5, and 8 ATM) throughout the procedure. The balloon catheter was paired with the Dolphin Inflation Device (Perouse Medical, Ivry le Temple, France). This inflation device features a syringe volume of 30 mL and can reach a maximum pressure of 30 ATM. Dilation was performed at least 3 times for 180 s. Thereafter, 5 mL of MMC at a concentration of 0.4 mg/mL was injected submucosally at a depth of approximately 2–3mm in a circumferential pattern just adjacent to the radial incisions or along the margins of the stenosis. To perform submucosal injections, we used a 23-gauge (0.6 mm) × 240 cm Interject^TM^ Injection Therapy Needle Catheter (Boston Scientific Corporation, Spencer, IN, USA). This catheter is equipped with a distinctive star-shaped inner design, enhancing needle actuation even in complex anatomical structures. Additionally, it incorporates a smoothly beveled needle for easier injections and a user-friendly locking hub to streamline the procedural process. All procedures were performed by an expert thoracic surgeon (G.F.). All patients were observed for a minimum of 24 h after the procedure. The postoperative treatment regimen comprised the following: Prednisone 25 mg daily for 10 days, with a gradual tapering schedule (starting at 25 mg/day for 4 days, followed by 12.5 mg/day for 3 days, and concluding with 5 mg/day for an additional 3 days), aerosol therapy with 1 vial of 800 µg/2 mL of beclomethasone suspension for nebulizer (Clenil^®^) administered twice daily for one month, and azithromycin 500 mg daily for 10 days.

### 2.7. Post-Treatment Follow-Up

A flexible bronchoscopic examination was scheduled in all patients 1 month after surgery or performed earlier if clinically indicated due to the presence of symptoms. Next, clinical data regarding follow-up were collected through quarterly outpatient consultations.

### 2.8. Statistical Analysis

All data were collected and stored in a Microsoft Excel^®^ spreadsheet. Dichotomous variables were reported as counts and percentages, while continuous variables as mean and standard deviation (SD), or as median and IQR (interquartile range) if the values were not normally distributed at the Shapiro–Wilk normality test. Statistical analyses were performed using the R software for statistical computing (R version 4.2.3, Foundation for Statistical Computing, Vienna, Austria). A value of *p* < 0.05 was considered to indicate statistical significance.

## 3. Results

### 3.1. Demographic Data

Sixteen patients, with an average age of 51.25 (±13.94) years, were eligible for the study, and among them, 8 (50.0%) were females. The causes of LTS varied: 6 patients (37.50%) had idiopathic stenosis, 5 patients (31.25%) developed LTS due to prolonged intubation, and the remaining 5 patients (31.25%) had LTS resulting from previous tracheostomy. Notably, COVID-19 was responsible for prolonged intubation and/or tracheostomy in 5 patients. None in the group were current smokers, but 5 (31.25%) patients were former smokers. The average BMI was 28.09 (±6.61). A significant majority of these patients (75.0%) had comorbidities, including high blood pressure, arrhythmia, diabetes, asthma, and chronic obstructive pulmonary disease, among others. A significant proportion of patients (75.0%) had a history of previous treatments. Specifically, 4 (25.0%) patients had undergone tracheal resection with end-to-end anastomosis, while 10 (62.50%) patients had undergone various endoscopic procedures. These endoscopic procedures included tracheal dilatation using a tracheobronchial balloon and/or laser radial incision through the stenosis, with or without the injection of corticosteroids, as well as granuloma resection, thermocoagulation, and the placement of a tracheal stent. Patients and treatment characteristics are shown in Table 1.

### 3.2. Bronchoscopic and Radiological Findings

Tracheal stenosis patterns, as assessed during flexible bronchoscopy, were predominantly found to be concentric (75.0% of cases). In 9 out of the 16 patients, a CT scan was deemed necessary. Subglottic stenosis was the most common location (88.90%), with 44.4% of patients classified as having grade 3–4 stenosis. The median length of the stenotic segment was 2.30 cm (IQR 2.10–2.50). On average, the distance from the vocal folds was 1.85 ± 1.16 cm. The median area of stenosis measured 58.93 mm^2^ (IQR 50.33–70.42).

### 3.3. Treatment Outcomes

**Figure 1:**
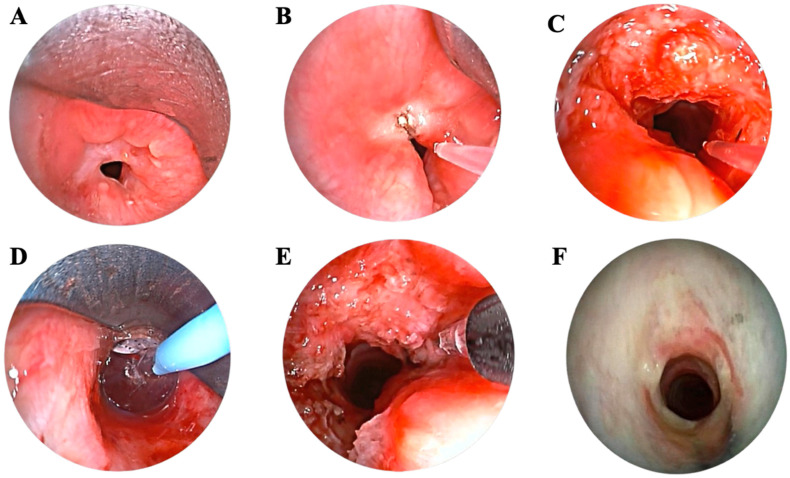
Different phases of endoscopic treatment of laryngotracheal stenosis. (**A**) Preoperative (**B**) During laser radial incisions (**C**) After radial laser incisions (**D**) Balloon dilation (**E**) Submucosal mitomycin C injection (**F**) At 1 month follow-up.

All patients underwent uneventful endoscopic treatment with no intraoperative complications or the need for conversion to open surgery. The standard endoscopic treatment used for all patients in the study is illustrated in Figure 1, with the outcomes at one month shown for one patient as an example. The average duration of the operation was 45.87 min (±13.72), and there were no complications during the postoperative period. While all index procedures were completed with successful restoration of a patent airway with symptom relief without emergent intervention, six patients experienced restenosis during follow-up and required additional treatments, with a median time to recurrence of 136.5 days (±43.9). Specifically, two patients were managed with endoscopic laser radial incision, tracheal dilation, and intralesional steroid injection. Three patients underwent the same endoscopic approach combined with MMC infiltration; one of these subsequently required tracheal resection due to persistent restenosis. The remaining patient underwent multiple endoscopic procedures with MMC infiltration and later silicone stent placement, followed by additional endoscopic revisions consisting of laser incision, dilation, and steroid injection. Details of individual recurrence patterns and subsequent interventions are summarized in Table 2. At last follow-up, all included patients, except for two who reported mild dyspnea during exercise, were completely asymptomatic. The median follow-up duration was 14.5 months (IQR 10.6–18.2).

## 4. Discussion

The development of benign tracheal stenosis is a complicated process involving various factors including immunity, inflammation, cell behavior, and oxidative stress [29,30]. In particular, the inflammatory response plays a crucial role. After a tracheal injury, persistent inflammation triggers the release of mediators such as interleukin (IL)-6 and IL-8, along with pro-fibrotic cytokines such as transforming growth factor β1 (TGF-β1) and vascular endothelial growth factor (VEGF). These factors drive repair processes, fibroblast activation and proliferation, suppression of apoptosis, extracellular matrix deposition, and disrupt the balance of collagen synthesis and degradation [29], ultimately leading to the excess granulation tissue responsible for tracheal stenosis.

Mitomycin is a naturally occurring antibiotic derived from the bacteria *Streptomyces caespitosis*, first isolated in 1956 [14]. It exists in various forms, with the c-form (MMC) being an alkylating agent that inhibits DNA synthesis through crosslinking, particularly during the late G1 and S phases. At higher concentrations, it can also disrupt RNA and protein synthesis [15].

The use of MMC in wound healing was initially suggested in 1963 for pterygium surgery [16] and has since been proposed in various ophthalmologic procedures, including glaucoma and corneal surgeries, treatment of corneal and conjunctival carcinomas, ocular cicatricial pemphigoid management, dacryocystorhinostomy, and optic nerve sheath fenestration. Studies investigating the effects of MMC on wound healing have provided insights into its mechanisms. Although the exact mechanism of action remains unclear, MMC appears to preferentially bind promoter sites of inducible genes [31,32], inhibiting their expression [33]. In rat models of cutaneous wounds, topical MMC application reduced the expression of mRNA for extracellular matrix proteins, leading to less wound fibrosis [34]. In cell culture experiments with human dermal fibroblasts, MMC increased the production of TGF-β1 and bFGF without causing cell death, which inhibited collagen production and promoted cell survival [35]. Similarly, studies involving keloid fibroblasts have shown that MMC reduces fibroblast density for about three weeks before the recovery of DNA synthesis and increased cell count occurred [36].

Preliminary studies have investigated the impact of MMC dosage, treatment duration, and delivery method in the context of cutaneous wound healing in both animal and human models [37,38,39]. In a rat model of cutaneous wound healing [37], intradermal MMC injections caused fibroblast apoptosis and reduced collagen and fibronectin levels, with similar outcomes across different concentrations used, suggesting that intradermal delivery may allow prolonged effects on wound healing compared to topical application. In this model, MMC was administered either by intradermal injection or topically at the time of injury. Wounds that received intradermal injections developed visible skin necrosis, while wounds treated topically exhibited mild fibrosis at 2 to 4 weeks post-injury. By the 6-month mark, old collagen was present in these wounds, similar to the control group.

Collectively, these studies suggest the existence of a critical time window during the wound healing process for optimal MMC effectiveness, with MMC delaying the wound healing response without entirely suppressing it.

Animal models were also used to investigate the use of MMC in airway injuries, particularly in laryngotracheal injury in dogs [18,40,41,42,43,44,45], rabbits [45,46,47,48,49], pigs [50], and ferrets [19]. In one of the earliest investigations, a canine model was employed to simulate acute subglottic injuries using cautery [40]. Endoscopic examinations and percentage assessments of airway patency indicated marked improvements in the MMC-treated dogs. In a separate study [44] on CO_2_ laser-induced subglottic stenosis, MMC-treated dogs displayed a statistically significant difference in the percentage of relative airway, with reduced collagen content in the wounds. Importantly, this study did not observe any complications associated with the high MMC concentration. However, when used in rabbit models, MMC yielded mixed results on airway injuries, with one study showing improvement in posterior glottic injury outcomes [47], while others investigating posterior subglottic and tracheal injury did not show favorable effects [46,47,48].

In 1998, MMC was first employed in the airway for the treatment of five pediatric patients facing severe recurrent tracheal granulation and scarring following tracheal reconstruction [51]. This approach, which involved the use of topical MMC in combination with bronchoscopy and laser treatment, appeared to diminish fibrosis and facilitate re-epithelialization before the formation of scar tissue. Encouragingly, all five patients were ultimately successfully decannulated. Since this pioneering use of MMC, numerous studies have explored its effectiveness in managing LTS [20,21,22,23]. However, these studies have yielded mixed results.

Assessing the benefits and risks of MMC in laryngotracheal stenosis treatment presents a challenge due to the absence of standardized dosages, durations, or application methods across studies. Research has reported a wide range of MMC dosages, spanning from 0.1 mg/mL to 10 mg/mL, coupled with exposure durations varying from 1 to 5 min [20,21,22,23]. Some researchers argue that higher MMC concentrations may be more effective in preventing scar tissue formation [52], possibly because higher doses or longer exposure times irreversibly inhibit fibroblast proliferation [52,53,54]. The method of MMC application in laryngotracheal stenosis treatment may significantly impact outcomes and consequently, the ability to draw definitive conclusions regarding its efficacy. While most studies rely on topical MMC application using cotton pledgets, this approach raises challenges in precisely quantifying the MMC penetration into the tissue. Consequently, the varying amounts of MMC reaching the affected area can lead to inconsistent results among patients. This lack of standardization in MMC dosage and delivery methods hinders the establishment of a clear, uniform treatment protocol for laryngotracheal stenosis.

In our study, we assessed 16 patients who underwent endoscopic treatment with MMC submucosal injection through a specific needle. All patients received effective treatment without experiencing any surgical complications or requiring open surgery. Nevertheless, we observed restenosis in six cases, necessitating further treatments, with a median recurrence time of approximately 136.5 days. However, after a median follow-up of 14.7 months, nearly all patients remained symptom-free, with only two reporting mild exercise-related dyspnea.

Recurrence following endoscopic treatment of laryngotracheal stenosis (LTS) remains a major challenge, with reported rates ranging from 40 to 100%, depending on etiology, stenosis length, and treatment modality [24]. Given these substantial recurrence rates, much of the literature has focused on whether adjunctive interventions can effectively reduce the risk of restenosis. Among these, corticosteroids and MMC have been most frequently investigated, although the results remain heterogeneous [20,21,22,23,24,25]. With regard to MMC, the most rigorous evidence derives from prospective randomized controlled trials. In a 2020 RCT, Yung et al. found no significant differences in symptom improvement, procedure-free interval, or spirometric outcomes between patients treated with topical MMC and those receiving placebo, suggesting no clear additive benefit [25]. Conversely, another randomized trial by Smith et al. reported that two applications of MMC delayed restenosis compared with a single application; however, 5-year recurrence rates were ultimately comparable, indicating only a transient effect [24]. Retrospective series have described longer symptom-free intervals and higher procedural success rates in MMC-treated patients, but these findings are limited by heterogeneous protocols and inherent selection bias [20,22,23]. Although the overall evidence remains inconsistent, the American College of Chest Physicians—acknowledging the low certainty and high risk of bias within available data—nonetheless endorses the use of local adjuvant therapy, including MMC, given the apparent prolongation of the interval to reintervention [10]. However, while most studies have focused on topical MMC application, alternative delivery methods may overcome some of its limitations. In this context, although topical application of mitomycin-C using pledgets is well described in the literature and has been incorporated into various endoscopic protocols for laryngotracheal stenosis, reports describing intralesional submucosal injection of MMC remain comparatively limited.

To the best of our knowledge, to date, only a single case series [55] and small number of case reports [56,57] have specifically evaluated this technique, including the 10-patient series by Tiran et al., in which submucosal injection was employed to facilitate more uniform MMC delivery during flexible bronchoscopy. In that study, the majority of patients reported satisfactory symptom improvement following treatment. In line with this rationale, our approach also utilizes submucosal MMC injection, which allows for a controlled, localized, and standardized delivery of the drug directly into the cicatricial tissue, potentially enhancing antifibrotic effect while avoiding the variability associated with topical application.

Furthermore, the precise quantification of MMC penetration into the tissue may help avert potential life-threatening complications. Notably, a retrospective review identified cases in which fibrinous debris obstructed the airway at the MMC application site, occurring in 5% of cases [24]. As previously mentioned, MMC’s ability to inhibit fibroblast proliferation can become irreversible with higher doses or prolonged exposure, ultimately leading to fibroblast cell death [53,54]. This, in turn, causes the accumulation of fibrinous exudate at the wound site, potentially leading to acute airway obstruction and contributing to additional scar tissue formation as the fibrinous exudate transforms into well-vascularized connective tissue. Pediatric patients may be at higher risk for this issue, especially if they lack a tracheostomy, owing to the smaller caliber of their airway. However, since airway complications related to topical MMC have also been observed in cases involving lower-dose preparations, it is likely that additional factors, not necessarily linked to MMC application, influence patients’ susceptibility to developing adverse events. Additionally, a recent case report documented the development of laryngeal cancer in a patient who had previously undergone topical MMC treatment for an anterior glottic web 2.5 years earlier [58]. Although the patient was a nonsmoker, a history of chronic laryngitis, vocal polyps, and keratosis was noted. To date, no other cases of delayed malignant transformation have been reported. Notably, in our study, we did not encounter any postoperative complications.

While our findings suggest that MMC is a viable and safe option for the endoscopic treatment of LTS, it is crucial to acknowledge several limitations of our study. Firstly, its retrospective design may introduce inherent biases and limitations associated with data collection. Additionally, the relatively small sample size might affect the generalizability of our results. The heterogeneity within our sample, including variations in previous treatments, multiple etiologies, and differences in lesions chronicity, could serve as potential confounding factors that could impact the interpretation of our findings. Furthermore, the absence of a control group hinders our ability to make direct comparisons and draw more definitive and firm conclusions about the advantages of using MMC in LTS treatment. Therefore, further prospective comparative studies, particularly randomized controlled trials, are needed to provide a more comprehensive assessment of MMC’s effectiveness. Additionally, trials assessing different MMC dosages would help to better understand its safety and efficacy in treating LTS. Cost-analysis studies could also offer valuable insights into the economic feasibility of this approach, contributing to a more comprehensive evaluation of its clinical value.

## 5. Conclusions

MMC appears to represent a viable and secure adjunctive measure in the endoscopic management of LTS. The submucosal injection of MMC may enhance precision in assessing tissue penetration and, consequently, yield more consistent results. Nevertheless, the essential need for further prospective comparative investigations, and ideally, randomized controlled trials, persists. These future studies would serve to provide a more comprehensive evaluation of MMC’s effectiveness within this clinical context and contribute to the establishment of a standardized treatment protocol.

## Figures and Tables

**Table 1:** Characteristics of the included patients. Abbreviations: BMI, body mass index; FU, follow-up; AF, atrial fibrillation; HTN, hypertension; HCE, hypercholesterolemia; COPD, chronic obstructive pulmonary disease; GERD, gastroesophageal reflux disease; CONC, concentric; ECC, eccentric; ELS, European Laryngological Association; N/A, Not Available.

Age (y)/Sex	Comorbidities	BMI	Smoker	Etiology	Intubation (d)	Trach.	Re-trach.	COVID-19 (Y = yes, N = no)	Previous Treatment	Site	Grade	ELS Classification	L (mm)	D (mm)	Pattern	Complications	Time to Recur. (d)	Further Treatments	Status	FU (d)
78/F	AF, HTN	36.1	No	Idiopathic	N/A	None	None	N	endoscopic procedures (*n* = 2)	Subglottic	N/A	N/A	N/A	N/A	CONC	None	129	stent (*n* = 1), endoscopic procedure (*n* = 2)	Dyspnea during exercise	924
69/M	HTN, diabetes, HCE	29.4	Ex	Idiopathic	N/A	None	None	N	None	Subglottic	III	IIIa	24	9	ECC	None	143	endoscopic procedure (*n* = 1), tracheal resection (*n* = 1)	No sympt.	731
48/M	HTN, HCE	29.8	No	Post-tracheostomy	42	None	None	Y	tracheal resection with end-to-end anastomosis (*n* = 1)	Subglottic	III	IIIa	22	23	CONC	None	N/A	None	No sympt.	461
48/M	None	19.6	Ex	Prolonged intubation	N/A	None	None	N	endoscopic procedures (*n* = 2)	Cervical trachea	II	IIa	19	32	CONC	None	207	endoscopic procedure (*n* = 1)	No sympt.	455
39/M	None	25.8	No	Prolonged intubation	15	None	None	Y	endoscopic procedures (*n* = 2)	Subglottic	N/A	N/A	N/A	N/A	CONC	None	N/A	None	Dyspnea during exercise	392
43/F	Ankylosing spondylitis	22.1	No	Idiopathic	N/A	None	None	N	endoscopic procedure (*n* = 1)	Subglottic	N/A	N/A	N/A	N/A	CONC	None	174	endoscopic procedure (*n* = 1)	No sympt.	430
62/M	HTN	27.2	No	Prolonged intubation	6	None	None	N	endoscopic procedure (*n* = 1)	Subglottic	II	IIa	44	0	CONC	None	N/A	None	No sympt.	742
50/F	None	32.8	No	Post-tracheostomy	N/A	Percutaneous	Surgical	Y	tracheal resection with end-to-end anastomosis (*n* = 1), endoscopic procedure (*n* = 7)	Subglottic	III	IIIa	25	19	CONC	None	78	endoscopic procedures (*n* = 2)	No sympt.	452
27/F	Asthma	17.4	No	Idiopathic	N/A	None	None	N	None	Subglottic	II	IIa	23	13	CONC	None	N/A	endoscopic procedures (*n* = 1)	No sympt.	496
28/M	None	30.2	Ex	Prolonged intubation	28	None	None	N	endoscopic procedure (*n* = 1)	Subglottic	II	IIa	16	38	CONC	None	N/A	None	No sympt.	342
65/M	COPD	34.3	Ex	Post-tracheostomy	35	None	None	Y	endoscopic procedure (*n* = 1)	Subglottic	III	IIIa	26	20	ECC	None	N/A	None	No sympt.	614
46/F	GERD, hypothyroidism	23.7	No	Idiopathic	N/A	None	None	N	endoscopic procedure (*n* = 1)	Subglottic	N/A	N/A	N/A	N/A	CONC	None	N/A	None	No sympt.	217
57/F	Asthma	19.9	No	Idiopathic	N/A	None	None	N	None	Subglottic	II	IIa	21	13	ECC	None	N/A	None	No sympt.	409
44/M	HTN	25.1	No	Prolonged intubation	Unknown	None	None	Y	tracheal resection with end-to-end anastomosis (*n* = 1), endoscopic procedure (*n* = 2)	Subglottic	N/A	N/A	N/A	N/A	CONC	None	130	endoscopic procedure (*n* = 1)	No sympt.	308
56/F	HTN, GERD	39.3	Ex	Post-tracheostomy	Unknown	Surgical	Surgical	N	T tube (*n* = 1), endoscopic procedures (*n* = 1)	Subglottic	N/A	N/A	N/A	N/A	ECC	None	N/A	endoscopic procedure (*n* = 1)	No sympt.	181
60/F	HTN, asthma, diabetes	36.7	No	Post-tracheostomy	Unknown	None	None	Y	tracheal resection with end-to-end anastomosis (*n* = 1)	Cervical trachea	N/A	N/A	N/A	N/A	CONC	None	N/A	endoscopic procedure (*n* = 1)	No sympt.	224

**Table 2:** Overview of recurrence intervals and further treatment modalities following initial endoscopic management of laryngotracheal stenosis.

Patient (Age/Gender)	Time to Recurrence (d)	Further Treatment
78/F	129	MMC injection + tracheal stent placement (*n* = 1); Laser-assisted radial stenosis release + dilation + steroid injection (*n* = 1)
69/M	143	Laser-assisted radial stenosis release + dilation + MMC injection (*n* = 1)
48/M	207	Laser-assisted radial stenosis release + dilation + steroid injection (*n* = 1)
43/F	174	Laser-assisted radial stenosis release + dilation + steroid injection (*n* = 1)
50/F	78	Laser-assisted radial stenosis release + dilation + MMC injection (*n* = 2)
44/M	130	Laser-assisted radial stenosis release + dilation + MMC injection (*n* = 1)

## Data Availability

The data presented in this study are available on request from the corresponding author.
